# Feedback in medical education – a workshop report with practical examples and recommendations

**DOI:** 10.3205/zma001339

**Published:** 2020-09-15

**Authors:** Christian Thrien, Götz Fabry, Anja Härtl, Claudia Kiessling, Tanja Graupe, Ingrid Preusche, Susanne Pruskil, Kai P. Schnabel, Monika Sennekamp, Stefan Rüttermann, Alexander Wünsch

**Affiliations:** 1 University of Cologne, Cologne Interprofessional Skills Lab and Simulation Center, Cologne, Germany; 2 Albert Ludwig University of Freiburg, Abt. für Med. Psychologie und Med. Soziologie, Freiburg im Breisgau, Germany; 3 University of Augsburg, Medical Faculty, Chair for Medical Didactics and Education Research, Augsburg, Germany; 4 LMU Munich Hospital, Institute for Medical Didactics and Education, Munich, Germany; 5 Witten/Herdecke University, Faculty of Health, Lehrstuhl für die Ausbildung personaler und interpersonaler Kompetenzen im Gesundheitswesen, Witten, Germany; 6 University of Veterinary Medicine Vienna, Assessment and Quality Assurance, Vienna, Austria; 7 University Medical Center Hamburg-Eppendorf, Department of General Practice and Primary Care, Center for Psychosocial Medicine, Hamburg, Germany; 8 University of Bern, Institute for Medical Education, Department for Education and Media, Bern, Switzerland; 9 University Hospital Frankfurt, Institute of General Practice, Frankfurt, Germany; 10 Goethe University Frankfurt, Carolinum Dental University Institute gGmbH, Poliklinik für Zahnerhaltung, Frankfurt/Main, Germany; 11 Technical University Munich, TUM Medical Education Center TUM MEC, Munich, Germany; 12 Cancer Center Freiburg - CCCF, Psycho-social Cancer Counselling in cooperation with the University Hospital Freiburg, Clinic for Psychosomatic Medicine and Psychotherapy, Freiburg, Germany; 13 University of Freiburg, Faculty of Medicine, Freiburg, Germany

**Keywords:** feedback, pschological, formative feedback, models, educational

## Abstract

**Background: **As a teaching method, feedback is an integral part of medical education. However, there is a lack of a uniform theoretical basis or generally recognized guidelines for its specific design. Against this background, the aim of this article is to discuss conceptual considerations and empirical findings regarding feedback using various practical examples.

**Procedure and conceptual considerations: **Building on the results of a workshop of the Committee for Communicative and Social Competences of the Society for Medical Education (GMA), this article first explains central conceptual considerations and empirical results on the topic of feedback. A particular focus is on various variables that influence the effect of feedback. This includes the feedback source, the frequency of feedback, starting points of feedback, the connection between feedback and reflection as well as the motivation and meta-cognitive skills of the feedback recipient.

**Practical examples: **The implementation of feedback in practice is illustrated using eight examples from the field of medical and dental education. They stem from various settings and the focus is on formative oral feedback. It will become evident that the focus is more on the givers of feedback than the recipients of feedback. Instructions for recipients of feedback on how to reflect on it is still the exception.

**Discussion: **Many of the relevant aspects for the effect of feedback described in the literature are already taken into account in the practical examples discussed. In conclusion, seven recommendations are made for implementing feedback in practice.

## Zusammenfassung

**Hintergrund: **Feedback ist als Unterrichtsmethode integraler Bestandteil der Medizinischen Ausbildung. Jedoch mangelt es an einer einheitlichen theoretischen Basis oder allgemein anerkannten Guidelines für die konkrete Ausgestaltung. Vor diesem Hintergrund ist das Ziel dieses Artikels, konzeptuelle Überlegungen und empirische Erkenntnisse zum Thema Feedback anhand verschiedener Praxisbeispiele zu diskutieren.

**Vorgehen und konzeptuelle Überlegungen: **Aufbauend auf den Ergebnissen eines Workshops des Ausschusses für Kommunikative und soziale Kompetenzen der Gesellschaft für Medizinische Ausbildung (GMA) werden in diesem Artikel zunächst zentrale konzeptuelle Überlegungen und empirische Ergebnisse zum Thema Feedback erläutert. Besonderes Augenmerk liegt auf diversen Variablen, die die Wirkung von Feedback beeinflussen. Dazu gehören die Feedbackquelle, die Häufigkeit des Feedbacks, Anknüpfungspunkte des Feedbacks, der Zusammenhang von Feedback und Reflexion sowie die Motivation und metakognitive Fähigkeiten der Feedbackempfängerinnen und -empfänger.

**Praxisbeispiele: **Die Umsetzung von Feedback in der Praxis wird exemplarisch anhand von acht Beispielen aus dem Feld der medizinischen und zahnmedizinischen Ausbildung dargestellt. Sie entstammen diversen Settings. Der Schwerpunkt liegt auf formativem, mündlichem Feedback. Es zeigt sich, dass hier eher die Feedbackgebenden im Fokus stehen, als die Feedbackempfangenden. Eine Anleitung zur Reflexion für die Feedbackempfangenden ist noch die Ausnahme.

**Diskussion: **Viele der in der Literatur beschriebenen relevanten Aspekte für die Wirkung von Feedback werden in den besprochenen Praxisbeispielen bereits berücksichtigt. Abschließend werden sieben Empfehlungen zur Umsetzung von Feedback in der Praxis formuliert.

## Introduction

Feedback as a teaching method is an integral part of medical education today. There are hardly any learning objective catalogs that do not contain the topic “giving and receiving feedback” [1], [2], [3], [4], [5], [6], [7], [8]. 

Accordingly, there is also a wealth of literature that deals with the topic of feedback, however mainly Anglo-American literature. The effectiveness of feedback depends on a variety of factors, they include the context of the feedback, the content and type of the feedback, characteristics and behavior of the person giving the feedback and the person receiving the feedback and their relationship to each other.

In autumn 2013, the Committee for Communicative and Social Competences (KusK) of the Society for Medical Education (GMA) carried out a survey of all German-language medical faculties in Germany, Austria and Switzerland, including a question about whether and how feedback is used in teaching communicative and social competences [9]. The answers showed that the use of feedback in this teaching context is widespread in the German-speaking countries, that the structure varies greatly and there is still no uniform theoretical basis for feedback. Internationally, too, it can be stated that although there are a large number of pragmatic recommendations on the topic of feedback, there is little literature on the theoretical basis and hardly any empirical evidence to be found (e.g. [10], [11], [12]).

Against this background, the aim of this article is to discuss conceptual considerations and empirical findings regarding feedback using various practical examples. The intention is to create a link between theoretical considerations on the one hand and everyday practice on the other. Finally, recommendations for teaching practice are developed based on the above foundations.

## Procedure

The article was created on the basis of a feedback workshop organized by the GMA KusK committee in June 2014. The 30 participants came from 17 medical faculties in Germany, Switzerland and the Netherlands. Everyone had experience with the topic of feedback in medical and dental education and further education, especially in the area of communicative and social competences. 

Initially, six selected articles [13], [14], [15], [16], [17], [18], which illustrate the range of the current state of literature, were discussed by the participants in small groups. The articles dealt with

- the question of the culture of feedback in the sense of an ongoing process that should stimulate reflection [13],
- the connection between specific feedback, the subsequent reflection of the person receiving the feedback and use of the feedback for the further learning process [16],
- the cognitive-psychological mechanisms of reflection in terms of disrupting and adapting scripts and self-schemes [17],
- the influence of the perception of feedback (especially negative feedback) from both teachers and learners on the effectiveness of feedback [18],
- the importance of the regulatory focus (prevention/promotion) in the field of medicine on the effect of feedback [14], and
- the effectiveness of the specific technique of the sandwich feedback on performance [15].

Following the discussion of the literature, previously selected practical examples based on the literature were worked on in parallel groups and in some cases tried out in practice. Using the practical examples, it was possible to discuss various approaches to feedback against the background of the literature. 

In the following, the theoretical considerations of the literature studied are presented, supplemented by further studies on the subject of feedback on the one hand, and the results of the discussion of the practical examples on the other hand. 

## Conceptual considerations

### Definition of feedback

Based on a systematic literature search, van de Ridder et al. [8] found nine characteristics that appear regularly in feedback definitions:

1. content of feedback,
2. aim of feedback,
3. recipient of feedback,
4. form of feedback,
5. how information on which feedback is given is obtained/prepared,
6. source of information on which feedback is given,
7. giver of feedback,
8. communication conditions for the feedback,
9. contextual factors of the feedback situation.

On this basis, van de Ridder et al. defined feedback as “[...] specific information about the comparison between a trainee’s observed performance and a standard, given with the intent to improve the trainee’s performance.”

Feedback therefore requires a standard with which an observed performance is compared and the result of the comparison is communicated to the person receiving the feedback with the aim of improving future performance [8]. The standard with which the performance is compared can be defined very differently, e.g. as a horizon of expectation laid down in writing; through the performance of a reference group of colleagues or learners; through previous performance of the learners themselves; or through the opinion of the teachers about what the learners should be able to do. The quality and source of the standard can therefore vary; the criteria that define a standard can be objective or subjective, absolute or relative [8].

#### Variables that affect the effect of feedback

The widespread assumption that feedback has a fundamentally positive effect is not readily supported by the results of empirical studies [11], [12], [19]. Rather, it appears that there are a large number of variables that influence the effect of feedback. This includes, among other things, the source of the feedback; the relationship between the recipient and the giver of the feedback; the content of the feedback; the time and the current internal state of the person receiving the feedback, which is determined by various emotional, motivational and cognitive aspects. 

The results of the studies mentioned above which deal with the following dimensions as possible factors influencing the effect of feedback are presented below:

- feedback source and frequency,
- theoretical models on the effect of feedback,
- the relationship between feedback and reflection,
- the feedback recipient and their motivation.

#### Feedback source and frequency

As part of a systematic review, Veloski et al. [12] examined which feedback factors influence the clinical performance of physicians. The majority of the 41 studies included were able to demonstrate positive effects of feedback. The authors concluded that feedback is particularly effective when it is provided by an authoritative and credible source, given regularly over a longer period. In addition to technical expertise, a respectful approach to the person receiving feedback also plays a role in the credibility amongst other factors [17], [20]. But Veloski et al. [12] also observed that there are relatively few results to date that have been obtained through randomised controlled studies. 

#### Models for the effect of feedback 

In a comprehensive review, Kluger and DeNisi [11] investigated the question of what positive or negative effect feedback has on individual performance. Based on their findings, they developed a model of feedback effect (Feedback Intervention Theory). They draw on a wealth of knowledge and concepts from motivational and cognitive psychology, but focusing primarily on the question of what a person focuses on when receiving and processing feedback.

With regard to the motivational and cognitive processes involved, they differentiate between three hierarchically related levels: The lowest level (task-learning processes) describes the processes that are necessary to complete a task, e.g. the chosen problem-solving strategy and the hypotheses contained therein about the solution of a task. The middle level (task-motivation processes) mainly describes effort management. In the event of a discrepancy between performance and standard, the effort is increased until the discrepancy is eliminated. Finally, the top hierarchical level (meta-task processes) includes processes that serve self-regulation, e.g. regarding self-image. If, for example, increased effort cannot achieve improved performance, then it is decided at the highest level whether it is worthwhile to continue attempts to complete the task. Whether the feedback source itself is credible enough to make further efforts worthwhile is also assessed at this level. 

These assumptions explain the different effects of feedback: The attention of a potential recipient of feedback is frequently directed at the middle level (task-motivation processes), because on the one hand many tasks are dealt with automatically and the focus is rarely on oneself on the other hand. Feedback shifts the recipient’s focus either to the lower level (task-learning processes), which may lead to alternative, improved strategies for action; or to the upper level (meta-task processes), where it may impair self-confidence for example and provoke the corresponding defensive reaction. The higher the feedback and thus the “locus of attention” in this hierarchy, the less effectively performance is influenced [10], [11], [13], [17]. 

Another feedback model is proposed by Hattie and Timperley [10], who focus their review on the conditions that maximize the positive effects of feedback. They emphasize the importance of clear learning goals and specific feedback on the learning process. Feedback must therefore provide answers to three key questions: 

“Where am I going? (What are the goals?), How am I going? (What progress is being made towards the goal?), and where to next? (What activities need to be undertaken to make better progress?)”

They also differentiate four levels to which feedback can be related, similar to the three levels described by Kluger and DeNisi [11]:

1. the task,
2. the process for solving the task,
3. self-direction of the learner,
4. the learner’s self.

It is true at all levels that feedback which relates to specific tasks and contains suggestions for improvement or new learning goals produces stronger effects than unspecific praise or blame. They confirm the low effectiveness of feedback at the level of self.

Accordingly, feedback can be particularly effective if it relates to the process of fulfilling the task or to the self-monitoring of the learner in relation to the learning process. In the latter case, this depends particularly on the person receiving the feedback, including the willingness to seek and deal with feedback, the trust in the correctness of one’s own actions, the sense of self-efficacy and the attribution of success and failure.

Feedback that relates to the task itself can in turn be very effective if it also points to the solution process and self-regulation. In addition, the timing of feedback plays a role in that immediate feedback is more effective at the task level, especially if it is simple, while delayed feedback is more effective at the process level and for more difficult and complex tasks [10], [13].

The type of feedback also seems to have an important impact. In one of the few empirical studies on the effectiveness of different types of feedback, v. Ridder et al. showed that positively embedded feedback increases the satisfaction and self-efficacy of the feedback recipient [21]. The study did not provide any clear results regarding performance. The performance of the group that received positively embedded feedback was better than the performance of the group that had negatively embedded feedback, but this was true even before the feedback intervention.

#### Feedback and reflection

In an observational study, Pelgrim et al. [16] discuss giving and receiving feedback in general practice consultations with real patients. They analyzed the relationship between *specific* feedback, learner reflection and use of the feedback measured against the development of an action plan for future learning. They found that only *specific* feedback from the trainers is followed by learner reflection. The reflection in turn promotes the use of the feedback for the development of action plans.

Poole et al. tackled the question of what feedback reflection must be like for it to lead to changes at the performance level [17]. In their view, reflection begins when events occur that break expectations of how things usually develop or question the self-images of the people involved. In an ideal scenario, the resulting stimulus leads to a critical analysis of one’s own knowledge and self-perception. This stimulates thinking and learning processes that lead to the revision of self-concepts and scripts and thus to the integration of new knowledge or new skills. This must be done by the learners. In order for this to work, certain prerequisites are necessary [22]:

1. adequate self-assessment,
2. metacognitive skills, such as reflection of one’s own thoughts and feelings, in order to process information conveyed in feedback and to be able to use it for competence development, and
3. the regulation of potentially occurring troublesome emotions as a result of questioning a positive self-image.

It is the task of the teachers to create a safe environment and to cultivate a competent relationship i.e. a respectful and supportive relationship with the learners. This includes paying attention to the affective aspects on the part of the learners [17], [22]. This helps them to accept and process the irritating stimuli of their self-concept and action scripts positively.

#### Feedback recipients and their motivation

Another variable whose influence on the effectiveness of feedback has been examined is the so-called regulatory focus. From a motivational psychological point of view, this merges various strategies that ultimately serve to increase pleasant emotional states and to avoid unpleasant ones [23]. On the one hand this can happen by striving for or maintaining pleasant conditions (promotion focus) or by avoiding unpleasant conditions (prevention focus). Depending on the personality structure of the individual but also influenced by the type of task or goal, the action in its basic direction is directed more towards promotion or prevention. Kluger and van Dijk [14] investigated how the regulatory focus interacts with positive and negative feedback. 

Tasks that activate the prevention system are primarily necessities, commitments, and things that need to be done to avoid pain, while the promotion system includes things that are considered desires or longings, something that you do because a successful completion promises joy. Tasks with a promotion focus therefore require more zeal, creativity and openness; tasks with a prevention focus tend to require more vigilance, attention to details and compliance with rules. According to Kluger and van Dijk [14], the health system is a good example of a mix of promotion and prevention focus.

“Doctors, for example, are required to be aware of potential mistakes and errors and at the same time to think innovatively, to handle complex situations and to make relatively risky decisions.” (ibid.)

With the promotion focus, positive feedback increases motivation and performance, negative feedback, on the other hand, reduces both. Conversely, negative feedback increases the motivation and performance of the prevention focus, positive feedback, on the other hand, actually has a negative impact on both motivation and performance.

#### Interim conclusion

Even if an overall theory of feedback is (still) to be formulated, some statements about feedback can be taken as certain. The findings can be grouped according to the following criteria:

- focus on the feedback giver or the feedback recipient and the relationship between the two,
- content/material of the feedback,
- the type of feedback.

With regard to feedback practice, the question arises as to which of the variables described above must be taken into account in everyday routines and, if necessary, can also be influenced in a targeted manner. This is discussed below using practical examples.

## The practical examples

The eight practical examples presented at the KusK workshop show selection of the wide range feedback in use at German-speaking medical faculties. They all share the goal of optimizing a behavior in a concrete manner and to ensure a constructive learning environment. The course of action is far from homogeneous and is based on different preliminary considerations.

Attachment 1 shows an overview of the eight practical examples based on the criteria described above. All practical examples are described in detail in the appendices.

## Discussion of the practical examples against the background of the literature

In the following, the practical examples presented in the attachments are critically discussed on the basis of the variables derived from the conceptual considerations for effective feedback in teaching. 

### Feedback source and frequency 

There is great consensus in the literature that singular feedback events are not sufficient, but that a feedback culture is required [11], [13], [16], [17], [24] within which feedback is given and received regularly and repeatedly. Every effort to train both teachers and students in giving feedback, as can be seen in various practical examples (see attachment 2 *History and Feedback* , see attachment 3 *Lecturer Training* , see attachment 4 *Student Basic Course* , see attachment 5 *Train the Trainer* , see attachment 6 *Dentistry* ), is therefore to be welcomed. Approaches with repeated and, if necessary, successive feedback, such as in the example of *General Practice* (see attachment 7 ), point in the right direction. 

A form of feedback developed by an entire group of experts, such as in the example of *Emotions* (see attachment 8 ), could be particularly effective from the point of view of relevance and thus the expected acceptance of the feedback source. For peer feedback, which is also used frequently, the question arises whether, with a view to improving performance, it may profit the learner giving feedback more than the learner receiving feedback. 

#### Models on the effect of feedback

The demand that feedback should not relate to the self-concept in communication training is problematic insofar as communication is always closely linked to personality. In this instance, it is difficult to meet the demand to leave out the personality of the feedback recipient. It is therefore all the more important and difficult to underline the subjectivity of feedback with clear personal framed I-statements, an aspect to which special attention is paid in the example of *Feedback Training for Standardized Patients (SPs)* (see attachment 9 ).

One problem is that the feedback giver cannot readily assess the inner state of the feedback recipient at that very moment. This also applies to the focus of attention. In order to avoid negative reactions here, approaches in which the learners are not the focus of the feedback themselves could be promising, e.g. because feedback is not about their own remarks in a patient interview, but rather their assessments of reactions they saw in a video (see attachment 8 *Emotions* ). Since the given reactions are not their own actions and the feedback also refers to the specific behavior option, it can be assumed that the learners do not understand this as a criticism of themselves even if they deviate from the expert opinion. However, it would have to be checked whether this form of indirect feedback actually affects performance in the scenario of leading a conversation. 

The more clearly the task and the requirements are described, the easier it will be to make reference to a specific task or situation. The expectation of guideline-based treatment recommendations and comprehensible conclusions in differential diagnoses in patient reports (see attachment 7 *General Practice* ) seem to be a good example here, especially if feedback is used to ensure that specific feedback is given for each of the given learning objectives. Knowledge gaps can be compensated for by appropriate references to teaching materials and suggestions, as also described in the *Dentistry *example (see attachment 6 ).

It is also advisable to take the specific learning objectives into account during teacher training in dealing with assessment checklists and their scaling and to communicate these to the learners as in the example of* General Practice* (see attachment 7 ). The students’ teaching objectives are specifically included in the training for moderation of peer feedback as well (see attachment 5 *Train the Trainer* ). This should increase the likelihood that students’ focus of attention will be on the task and its accomplishment.

With regard to feedback, the type of task is important insofar as delayed feedback is more effective for knowledge transfer or for more complex tasks [13]. It is therefore also conducive to learning if learners, for example, first complete patient reports before receiving feedback (see attachment 7 *General Medicine* ).

#### Feedback and reflection

Clarifying learning goals is one thing, addressing deviations is another. When considering this, it is also beneficial to use stimulus material in feedback training that also contains behavior that is worth criticizing or that provokes conflicts, to stimulate reflection on it, as in the examples of the *Basic Student Course* (see attachment 4 ) and *Train the Trainer* (see attachment 5 ) or *SPs* (see attachment 9 ).

The more specific the feedback, the more it is used to develop new, individual learning goals or action plans upon reflection [25]. This can be supported by references to learning content that should already have been learned, as described in the *Dentistry *example (see attachment 6 ) or through additional feedback with a request to revise reports, as in the example of General Practice (see attachment 7 ).

Since the teachers in the field of medical education are often still learners in terms of feedback, their reactions which might lead to inhibitions must also be taken into account. The authors of the *Lecturer Training practical* example (see attachment 3 ) report less resistance when formulating feedback if the participants have initially developed the feedback rules themselves, compared to a previously practiced deductive approach. This could have to do with the fact that the self-concepts remain intact during the formulation of the feedback rules or that they are casually reconstructed or further developed.

Likewise, in the practical example of* Dentistry* (see attachment 6 ) it is reported that precisely those students who did not take part in the voluntary communication training, fall back on justifying themselves when given feedback, i.e. defending their self-concepts and scripts. In this light, it seems advisable to prepare teachers for the moderation of peer feedback, as described in the *Train the Trainer* example (see attachment 5 ). In addition to well-balanced learning objectives, instruction in reflection in terms of a reconstruction of self-concepts and scripts could be explicitly addressed.

Another option to limit the necessary irritation caused by feedback to a tolerable and beneficial level could be to allow students themselves to select the patient about whom they will report (see attachment 7 *General Practice* ). It can be assumed that students are likely to avoid cases which were particularly difficult for them and in which they would expect predominantly negative feedback. Written work samples on giving feedback (see attachment 5 *Train the Trainer* ) might also be a good option from this point of view, since written feedback offers the opportunity for optimization before it is submitted. There would therefore be high probability of receiving a rule-compliant work sample, so that modifications made to the feedback may be required to allow use of the compare and contrast method. In this way, negative examples can be used without being or having to be publicly attributable to an individual, which should reduce the likelihood of resistance to reflection.

#### Feedback recipients and their motivation

In order to prepare teachers and medical practitioners well for the challenge of accompanying lifelong learning processes, it is advisable to anticipate the different learner or colleague reactions in role play, as described in the example of* Lecturer Training* (see attachment 3 ) using modification of the reflection levels of the feedback recipients or colleagues. Three learner reflection levels are distinguished and represented in the role play:

1. reflected behavior with own solution ideas,
2. reflected behavior without own solution ideas,
3. no reflected behavior.

In this way, training does not remain at the quality level of the primary feedback message, but incorporates the learners’ reactions, from those conducive to learning to those inhibitory to it, and helps to develop constructive approaches.

The question of whether positive or negative feedback is more helpful becomes a complex problem against the background of the importance of the regulatory focus described above. In a feedback culture in which negative feedback is avoided [18], it is therefore useful to observe activities that learners tackle under the promotional focus as far as possible. In this scenario too, a degree of freedom in task selection or, as in the example of *General Practice* (see attachment 7 ), at least the patient who they will report on, is all the more crucial. The sub-task of recognizing and observing red flags (information that is indicative of a dangerous progression of an illness) could activate the prevention focus, at least if it is clearly communicated beforehand. With regard to the red flags, decidedly negative feedback should therefore also be given if these are overlooked.

#### Strengths and limitations

Limitations of the present work result from the way it was created. The literature used in the workshop and the practical examples presented was not selected on the basis of systematic, comprehensive literature research or on the basis of a theoretically sound concept. Rather, the authors involved in the selection process suggested current articles that seemed particularly relevant to them against the background of informally conducted current discussions. The selection of the practical examples presented was also not strictly theory-based and systematic. Instead, registered workshop participants and individual authors who intended to take part in the workshop were asked to submit practical examples. This pragmatic approach was justifiable in view of the preparation of a workshop with a limited number of participants and addressing the specific interests and questions of the participants.

The practical examples therefore certainly do not comprehensively cover the practice of feedback in medical education in Germany, Austria and Switzerland. However, the practical examples give a good insight into the corresponding teaching-learning situations that are used in medical education. The individual examples are always adapted to there faculty’s internal circumstances, resources and the individual setting. Based on the practical examples, interested parties can get stimulated by the variety of ideas and develop their own concepts.

The following recommendations can be derived from the theory and the practical examples discussed:

## Conclusion/recommendations

Based on the literature examined and the practical examples described above, various aspects can be derived for medical education. Feedback is a complex process, the success of which depends on many variables, which overlap in individual cases and can inhibit or promote each other in their effects. Nevertheless, recommendations can be formulated that can have a positive effect on the effectiveness of feedback:

1. A learning and feedback culture is required that enables stable, respectful and supportive relationships between teachers and learners – in the sense of credible and authoritative feedback sources – and in which feedback is given regularly after sufficient observation of the learners.
2. Feedback must be integrated into teaching and learning processes. The standard against which they will be compared must be communicated transparently: what goals are to be achieved, how is this happening now and how can and should it continue? (Operation and action plan).
3. Feedback should mainly focus on the processes, how learners perform tasks and direct themselves. Feedback on the results of a task can effectively support this if it refers to the processes. Feedback that relates only to the person without reference to a specific action should be avoided.
4. People giving feedback must be trained to recognize the levels to which the feedback should be directed in accordance with point 3 of these recommendations and to address them in a targeted manner.
5. Feedback may and should be irritating in the sense of disturbing inadequate self-concepts and scripts. Such disturbed self-concepts and scripts of the learners have to be reconstructed through reflection. Teachers should enable these processes and support them respectfully, and constructively.
6. Feedback givers have to be prepared to support the reflection processes in order to promote constructive reactions of the learners. The regulatory focus and the reconstruction of self-concepts and scripts can be key concepts in this.
7. Learners must also be explicitly prepared for the reflection process in order to develop the metacognitive skills they need to perceive and use feedback as a learning opportunity.

## Acknowledgements

We would like to thank everybody who helped organize the workshop with support from the GMA. Special thanks to J.M. Monica van de Ridder for the thematic management of the workshop and many stimulating impulses. We thank the authors for the stimulating, diverse, exciting practical examples, especially Waltraud Silbernagel, Martin Perrig, Mireille Schaufelberger and Michaela Wagner-Menghin. And of course many thanks to all participants in the KusK workshop for open, interested, benevolently critical and constructive discussions.

## Competing interests

The authors declare that they have no competing interests. 

## Supplementary Material

Table 1: Overview of the practical examples

Practical Example Patient History and Feedback

Practical Example Lecturer Training

Practical Example Student Basic Course

Practical Example Train the Trainer

Practical Example Dentistry

Practical Example General Practice

Practical Example Emotions

Practical Example Standardized Patients (SPs)
