# Supplementary material for: Feedback in medical education – a workshop report with practical examples and recommendations
Source: GMS J Med Educ. 2020 Sep 15;37(5):Doc46. doi: 10.3205/zma001339 (PMC7499466; doi:10.3205/zma001339)
Supplement: Table 1: Overview of the practical examples [file JME-37-46-s-001.pdf]

Table 1: Overview of the practical examples

Example (Authors)	General description<br>1. Aim<br>2. Setting<br>3. Target group<br>4. Procedure/context	Focus on giver vs. recipient and relationship between the two	Content/material of the feedback	Type of feedback
**Patient History and Feedback (Perrig M, Schaufelberger M, Schnabel K)**	1. Take a structured patient history, give adequate, constructive feedback, be open to receiving feedback<br>2. Pre-clinical Phase<br>3. Students<br>4. Combination of feedback training with training in taking patient history	• Focus on feedback giver<br>• Students amongst themselves<br>• Teaching staff to students	Role play in groups of three on a doctor-patient consultation with changing roles (doctor as feedback recipient, patient, observer as feedback giver) and the task of taking a simple patient history.	• Formative<br>• Oral
**Lecturer Training (Härtl A, Kiessling C)**	1. Provide constructive feedback regardless of hierarchy in everyday clinical practice<br>2. Further education<br>3. Lecturers<br>4. Feedback training in which feedback is also given	• Focus on feedback giver<br>• Teaching staff amongst themselves<br>• Doctors amongst themselves, regardless of hierarchies	Short instructional film with a discharge visit, for which the participants begin drafting written feedback. In role play, the level of reflection of the recipient is modified (reflected with own solution ideas; reflected without solution ideas; no reflection).	• Formative<br>• Written by participants<br>• Oral feedback for feedback
**Basic Course for Students (Fabry G, Silobernagel W)**	1. Giving constructive feedback<br>2. Pre-clinical Phase<br>3. Students<br>4. Feedback training in which feedback is also given	• Focus on feedback giver<br>• Students amongst themselves<br>• In role play, the recipient takes on the role of a doctor seen in a video	Instructional video showing a doctor-patient conversation that contains aspects that can be critically assessed.	• Formative<br>• Oral

Example (Authors)	General description<br>1. Aim<br>2. Setting<br>3. Target group<br>4. Procedure/context	Focus on giver vs. recipient and relationship between the two	Content/material of the feedback	Type of feedback
**Train the Trainer (Preusche I, Wagner-Menghin M)**	1. Giving students (behavior-based) feedback and moderating peer feedback<br>2. Clinical phase, further education<br>3. Lecturers<br>4. Feedback training in which feedback is also given	• Focus on feedback giver<br>• Lecturers amongst themselves to transfer to the feedback to students	Written, anonymized, partly modified feedback from the participating lecturers who gave the feedback in advance of training regarding a video viewing with doctor-patient contact. Compliant and non-compliant forms of feedback are presented (compare and contrast). The teaching goals for the students are included and a balanced consideration of the teaching goals is trained in the feedback.	• Formative<br>• Written in advance as an example<br>• Orally in training
**Dentistry (Rüttermann S and co-workers)**	1. Reflection of a treatment session regarding practical skills, theoretical knowledge and doctor-patient communication<br>2. Clinical phase<br>3. Students<br>4. Pure feedback giving in teaching situations	• Focus on feedback giver<br>• Tutor (dentist) to students	Behavior in treatment sessions of students with real patients. Practical skills, knowledge and communication are addressed.	• After initial summative feedback, switched to formative.<br>• Orally, in writing<br>• In the case of theoretical deficits, references to the learning platform, scripts, literature
**General Practice (Jansen P)**	1. Structure patient contacts, justify and document diagnosis and treatment<br>2. Clinical picture<br>3. Students<br>4. Pure feedback in teaching situations	• Focus on feedback giver<br>• Teaching staff to students	Patient reports written by students on two-week practice blocks in General Practice. These should show a structured approach from taking the patient's history, assessment of the findings, development of suspected and treatment diagnoses to treatment recommendations.	• Formative in the 3rd block<br>• Summative in the 4th block<br>• Written<br>• Oriented towards a written horizon of expectation<br>• Checklist and free text comments

Example (Authors)	General description<br>1. Aim<br>2. Setting<br>3. Target group<br>4. Procedure/context	Focus on giver vs. recipient and relationship between the two	Content/material of the feedback	Type of feedback
**Emotions (Graupe T, Kiessling C)**	1. Recognize open and hidden clues of concerns and show appropriate responses<br>2. Pre-clinical Phase<br>3. Students<br>4. Pure feedback in teaching situations	• Focus on feedback giver<br>• Indirectly from a group of experts to students	Videos with doctor-patient interactions, in which open or hidden clues to patient emotions play a role. The students assess the appropriateness of five alternative courses of action with which the doctors' might react to the information provided by the patients in the video. Based on their assessment, they receive feedback formulated by an expert group.	• Formative<br>• Written, pre-formulated
**Standardized Patients (SP) (Thrien C)**	1. Adequate and constructive feedback on leading a medical consultation and dealing with patients<br>2. Training of SPs<br>3. SPs<br>4. Feedback training in which feedback is also given	• Focus on feedback giver<br>• SPs amongst themselves<br>• SP trainer to SPs	Improvised interactions in a non-medical context, which are usually prone to conflict (e.g. customer with high expectations for advice meets inexperienced salesperson).	• Formative<br>• Oral

Example: The examples are listed in the order of the appendices with the detailed descriptions.
General Description: The aim describes the general objective of the event, which goes beyond the specific aim of the feedback.
Focus: As described in the text, training for feedback givers as well as feedback recipients makes sense but is not (yet) implemented everywhere, which is why this aspect is shown in the table.
Content/Material of the Feedback: Here the observable behavior is named, for which in the training feedback is given.
Type of Feedback: Basic parameters of the feedback in the event described are named here.
