# Supplementary material for: Feedback in medical education – a workshop report with practical examples and recommendations
Source: GMS J Med Educ. 2020 Sep 15;37(5):Doc46. doi: 10.3205/zma001339 (PMC7499466; doi:10.3205/zma001339)
Supplement: Practical Example Patient History and Feedback [file JME-37-46-s-002.pdf]

Attachment 2: Practical Example Patient History and Feedback

**Title/keyword of the training**	**Patient history and feedback training 1st year of study**
**Authors**	Dr. med. Martin Perrig, MME; Dr. med. Mireille Schaufelberger; Dr. med. Kai Schnabel, MME
**Institution**	Faculty of Medicine at the University of Bern
**Setting**	Part of the internship in the 1st academic year.
**Aim**	The aim of the internship is for students to master the structure of taking a patient's general medical history at the end of the course, give adequate constructive feedback and be open to receiving feedback.
**Feedback giver**	Students of human and dental medicine in the 1st academic year and experienced clinicians trained in feedback or communication experts
**Feedback receiver**	Students of human and dental medicine in the 1st academic year
**Feedback material**	Role-plays in which students take and observe a patient's medical history involving an uncomplicated illness.
**Manner of feedback**	Formative "positively framed" feedback from lecturers as well as peers. Then oral feedback about the feedback from lecturers.
**Procedure**	3 students each in a room go through three different roles in the role play (uncomplicated illnesses that everyone has had before: cold, headache, abdominal pain). 1st doctor, 2nd patient, 3rd observer and feedback provider. Each consultation lasts 10 minutes, after which feedback is given or received.<br>First, the interviewing students are asked to assess themselves overall, then they are asked to name what they have done particularly well, then the observers and the patient actor give positive feedback, and finally the lecturers. The whole procedure is then repeated again with constructive suggestions for improvement and the lecturers give feedback on the feedback and give motivating final overall feedback. After 20 minutes, the roles are rotated so that after one hour, everyone has played each role once.
**Rationale**	From the first year, students are in the General Practice internship. In the third year, they do Clinical Skills Training (CST), in which they have to take medical histories and physical examinations with real patients. From the master's degree (4th year) onwards, students receive feedback from SPs in communication training (Schaufelberger et al) and give feedback to each other. In order to prepare them for both roles, the patient history and feedback training was introduced, all appropriate for the student's level as part of the first year communication curriculum.

Attachment 2 to: Thrien C, Fabry G, Härtl A, Kiessling C, Graupe T, Preusche I, Pruskil S, Schnabel K, Sennekamp M, Rüttermann S, Wünsch A. *Feedback in medical education – a workshop report with practical examples and recommendations*. GMS J Med Educ. 2020;37(5):Doc46.
DOI: 10.3205/zma001339

**Literature**	Schaufelberger M, Frey P, Woermann U, Schnabel K, Barth J. Benefits of communication skills training after real patient exposure. Clin Teach. 2012;9:85-8.
**Experiences**	Very good, the students greatly appreciate the internship. The role of the doctor is very challenging for dental students. Since the two courses are identical in the first two years, this cannot be avoided.
**Further development**	So far only one change in lecturer training. Since it turned out to be unrealistic to conduct separate training sessions, the lecturers are now trained directly before the internship. The event was incorporated into the curriculum after a pilot run.

Attachment 2 to: Thrien C, Fabry G, Härtl A, Kiessling C, Graupe T, Preusche I, Pruskil S, Schnabel K, Sennekamp M, Rüttermann S, Wünsch A. *Feedback in medical education – a workshop report with practical examples and recommendations*. GMS J Med Educ. 2020;37(5):Doc46.
DOI: 10.3205/zma001339
