# Supplementary material for: Feedback in medical education – a workshop report with practical examples and recommendations
Source: GMS J Med Educ. 2020 Sep 15;37(5):Doc46. doi: 10.3205/zma001339 (PMC7499466; doi:10.3205/zma001339)
Supplement: Practical Example Lecturer Training [file JME-37-46-s-003.pdf]

Attachment 3: Practical Example Lecturer Training

**Title/keyword of the training**	**Feedback in everyday clinical practice**
**Authors**	Anja Härtl, Claudia Kiessling
**Institution**	University Hospital Munich, Institute for Medical Didactics and Education
**Setting**	Teacher training as part of a 2.5-day faculty development workshop; feedback is practiced as part of microteaching in small groups with teachers.
**Aim**	Participants learn to give constructive feedback to colleagues in everyday clinical practice regardless of their position in the hierarchy
**Feedback giver**	Teachrs in human medicine with teaching and assessment experience
**Feedback receiver**	Teachers in human medicine with teaching and assessmentexperience
**Feedback material**	Short educational vidoe (example of a discharge consultation)
**Feedback type**	Direct verbal feedback to a participant who takes on the role of the doctor from the educational video
**Procedure**	The inductive teaching concept is based on the AVIVA Model[1] for the development of teaching units. The unit lasts 45 minutes with a group of 8 to 36 participants and one or two facilitators.<br>Arrive and prep: Brainstorm where and when you might encounter feedback in a private and professional context<br>Activate prior knowledge: Examples of participants of positive feedback experiences – by paraphrasing the examples the facilitators note the first feedback rules on a flipchart<br>Inform: brief input on feedback/feedup/feedforward, goal orientation in the feedback process and the importance of feedback in teaching<br>Process: 90 sec video clip of a ward round with observation task; formulate feedback in 3 steps (1st collect feedback in teams of two, then as a group; 2nd prioritize; 3rd formulate the start of the feedback in new teams of two in writing)<br>Evaluate: Role play (3 rounds); conducting a feedback conversation, the feedback recipient takes on three different levels of reflection (reflected and own solution ideas; reflected and no solution ideas; not reflected); each round of feedback on feedback from the feedback giver, feedback recipient, group and facilitators – additional rules for feedback are generated<br>Finally, a summary by facilitators and transition to microteaching.
**Rationale**	The inductive teaching concept aims a highlighting to the participants right from the start that feedback is an individual process despite general rules. This is not only influenced by the feedback giver but also by the feedback recipient, who takes on a different level of reflection when instructed by the facilitators. The independent

	development of feedback rules supports the transfer into one's own actions. In role plays, the participants learn through self-awareness, observation and giving feedback on the feedback that has just been observed.
**Literature**	[1]Städeli, Ch., Grassi, A, Rhiner, K. & Obrist. W. (2010) Kompetenzorientiert unterrichten. Das AVIVA-Modell. Bern: hep-verlag
**Experiences**	Participants evaluated the inductive approach positively, the documentation on the flipchart is essential in order to visualize the ideas and rules developed for feedback. Compared to the previous lesson on feedback, which used a more deductive approach, there was significantly less resistance among the participants when it came to formulating feedback. Depending on the previous experience of the participants, fundamental rules for the feedback process should be worked out in more detail.
**Further development**	Identify aids that would allow quicker adaption of the feedback to the level of reflection of the other person.

Attachment 3 to: Thrien C, Fabry G, Härtl A, Kiessling C, Graupe T, Preusche I, Pruskil S, Schnabel K, Sennekamp M, Rüttermann S, Wünsch A. *Feedback in medical education – a workshop report with practical examples and recommendations*. GMS J Med Educ. 2020;37(5):Doc46.
DOI: 10.3205/zma001339
