# Supplementary material for: Feedback in medical education – a workshop report with practical examples and recommendations
Source: GMS J Med Educ. 2020 Sep 15;37(5):Doc46. doi: 10.3205/zma001339 (PMC7499466; doi:10.3205/zma001339)
Supplement: Practical Example Student Basic Course [file JME-37-46-s-004.pdf]

Attachment 4: Practical Example Student Basic Course

**Title/keyword of the training**	**Feedback training for students**
**Authors**	Götz Fabry<br>Waltraud Silbernagel
**Institution**	Faculty of Medicine at the Albert-Ludwigs University, Medical Psychology and Sociology
**Setting**	Medical Psychology Course, 1st academic year: The course is held in parallel groups of 12-14 students each. After an introduction, which serves to activate the previously taught basic consultation skills, the students conduct consultations with SPs. Each student has at least one consultation, for this they are divided into small groups (max. 4 participants).
**Aim**	In this part of the course, students should learn how to give constructive feedback. This should enable them to give each other feedback on their consultations as the course progresses.
**Feedback giver**	Medical students in the 1st academic year
**Feedback receiver**	Medical students in the 1st academic year
**Feedback material**	Teaching video with doctor-patient consultation (Source: Exercises for medical consultations, Film C 1937 "Leben im Risiko", IMF 1997).
**Feedback type**	Direct verbal feedback given to another student who takes on the role of the doctor seen in the video.
**Procedure**	First, basic feedback rules are worked out with the students on a flipchart. Then the students see the instructional video. Beforehand, they receive the instruction: "Please imagine that you are an intern in this practice and are asked by the doctor to give him feedback after the consultation. So think about what you like about the consultation, what you don't like and what you think could be improved."<br>At the end of the video, the students are divided into two groups, with the task of discussing their observations and assessments and developing a group feedback. This feedback must be given by a person from the group in a role play.<br>After about 20 minutes of group work, a role play takes place. For this purpose, one participant from each group gives feedback to the doctor (silent role, played by a participant from another group). Afterwards, the feedback will be reflected on as a group, first asking the "doctor" for their impression. Then the role play is repeated with the feedback from the other group and also reflected upon.
**Rationale**	After observing a doctor-patient consultation (or student-patient consultation), the students should be prepared to give appropriate and constructive feedback. Therefore, an example was chosen for the feedback training that is as close as possible to the later practice situation. The group work is intended to sensitize students to the fact

Attachment 4 to: Thrien C, Fabry G, Härtl A, Kiessling C, Graupe T, Preusche I, Pruskil S, Schnabel K, Sennekamp M, Rüttermann S, Wünsch A. *Feedback in medical education – a workshop report with practical examples and recommendations*. GMS J Med Educ. 2020;37(5):Doc46.
DOI: 10.3205/zma001339

	that a situation can be perceived and assessed very differently. Through role playing, students see two examples of formulating feedback that they can reflect on critically. The selected video contains many positive aspects but also some that can be evaluated critically.
**Experiences**	Overall, very good experiences. On the one hand, the students deal very intensively with various aspects of the doctor’s consultation skills, which leads to intense discussions in the groups. On the other hand, the implementation of the feedback through the group work is very successful.
**Further development**	We originally ran another feedback exercise right at the beginning in which the students were asked to give feedback to a lecturer from their semester about their course. However, since this did not bring any additional benefit, we have discontinued this exercise.

Attachment 4 to: Thrien C, Fabry G, Härtl A, Kiessling C, Graupe T, Preusche I, Pruskil S, Schnabel K, Sennekamp M, Rüttermann S, Wünsch A. *Feedback in medical education – a workshop report with practical examples and recommendations*. GMS J Med Educ. 2020;37(5):Doc46.
DOI: 10.3205/zma001339
