# Supplementary material for: Feedback in medical education – a workshop report with practical examples and recommendations
Source: GMS J Med Educ. 2020 Sep 15;37(5):Doc46. doi: 10.3205/zma001339 (PMC7499466; doi:10.3205/zma001339)
Supplement: Practical Example Train the Trainer [file JME-37-46-s-005.pdf]

Attachment 5: Practice Example Train the Trainer

**Title/keyword of the training**	**Feedback training for teachers in consultation skills, train the trainer**
**Authors**	Ingrid Preusche & Michaela Wagner-Menghin
**Institution**	Medical University Vienna
**Setting**	Teaching staff workshop – 3-hour training of existing and new teaching staff for the course (LVA) “Medical Consultation Skills A” (2-hour lessons over 6 weeks in the 3rd semester with role play with tutor actors and professional patient actors (3 units each) on the subject of taking patient history; video-based, 360° feedback)<br>Participants: approx. 40 teaching staff, who then teach the entire cohort of the 2nd year of study in approx. 72 small groups
**Aim**	Teaching staff are prepared to 1) give students (behavior-based) feedback on their consultation skills and 2) moderate peer feedback from students.
**Feedback giver**	Teaching staff in consultation skills (3rd semester)
**Feedback receiver**	Teaching staff in consultation skills (3rd semester)
**Feedback material**	Videotaped doctor-patient consultation. Written, anonymized, partially modified feedback that the participants give in advance of the training on a video recording of a doctor’s consultation.
**Feedback type**	Analysis of written, anonymized, partially modified feedback, which the participants give in advance of the training on a video recording of a doctor’s consultation
**Procedure**	1) The participants as trainers for the LVA “Medical Consultation Skills A” submit a written work sample of “giving feedback” as part of their assignment. This should involve them watching a video that shows a doctor’s consultation and give the “doctor” written feedback. “Online task”, estimated workload: 15 minutes<br><br>2) In the actual workshop on the topic of feedback, the written feedback from the participants is collected in advance, anonymized and changed slightly. The examples of feedback are then discussed:<br>Feedback examples for activity 1: Illustration of compliant vs. non-compliant feedback design, whereby the feedback rules “direct, concrete (with examples), I-message, value-free, positive and negative points” are given as a guideline (described in the freely available teaching materials for students and teachers, which can be downloaded at any time.)<br>Feedback examples for activity 2: Illustration of balanced vs. unbalanced reference of the feedback to the teaching objectives of the LVA (consultation content, style and relationship/contact with the patient). These examples in 2 were all designed in accordance with

Attachment 5 to: Thrien C, Fabry G, Härtl A, Kiessling C, Graupe T, Preusche I, Pruskil S, Schnabel K, Sennekamp M, Rüttermann S, Wünsch A. *Feedback in medical education – a workshop report with practical examples and recommendations*. GMS J Med Educ. 2020;37(5):Doc46.
DOI: 10.3205/zma001339

	the rules in order to focus on the uniform representation of the teaching objectives in the feedback in activity 2.<br><br>3) In the workshop, "Examples 1" are first read in small groups and their quality is discussed. Afterwards, comments are collected from the small groups and discussed in the large group. In conclusion, the teaching requirement ("feedback rules", with reference to the teaching material) is clearly communicated.<br>The procedure is then repeated with "Examples 2".
**Rationale**	Giving feedback is a key aspect of medical education. Standardization and further development of the teaching staff's skills in giving feedback is therefore an important didactic measure.<br><br><u>Rationale for activity 1:</u> Teaching staff identify and develop characteristics of feedback rules themselves using best practice and worst practice examples ("compare & contrast") so that they can better reflect on their own feedback practice in the future.<br><br><u>Rationale for activity 2:</u> Teaching staff work out different aspects of balanced feedback using best practice and worst practice examples ("compare & contrast") so that they can better reflect on their own feedback practice in the future.
**Literature**	Kurtz S, Silverman J, Draper J. *Teaching and Learning Communication Skills in Medicine.* 2nd ed. Oxford: Radcliffe Publishing; 2005.<br>Silverman J, Kurtz S, Draper J. *Skills for Communicating with Patients.* 2nd ed. Oxford: Radcliffe Publishing; 2005.
**Experiences**	By including the (anonymized) texts of the teaching staff in the workshop materials, all workshop participants were actively involved. Feedback was perceived as an important topic and the learning goals were clearly communicated:<br>Students can competently take a patient's history (obtain the required information in a patient-centered manner)<br>Students are able to give and accept feedback.
**Further development**	Working through the same material a second time is no longer useful for "experienced" teachers.<br>Modification: new stimulus material<br>Modification discussed: Provide suggestions for collegial feedback on your own feedback practice and organize the structure for it.

Attachment 5 to: Thrien C, Fabry G, Härtl A, Kiessling C, Graupe T, Preusche I, Pruskil S, Schnabel K, Sennekamp M, Rüttermann S, Wünsch A. *Feedback in medical education – a workshop report with practical examples and recommendations*. GMS J Med Educ. 2020;37(5):Doc46.
DOI: 10.3205/zma001339
