# Supplementary material for: Feedback in medical education – a workshop report with practical examples and recommendations
Source: GMS J Med Educ. 2020 Sep 15;37(5):Doc46. doi: 10.3205/zma001339 (PMC7499466; doi:10.3205/zma001339)
Supplement: Practical Example Dentistry [file JME-37-46-s-006.pdf]

Attachment 6: Practical Example Dentistry

**Title/keyword of the training**	**Feedback as part of courses in dental treatment**
**Authors**	Rüttermann, S. and co-workers
**Institution**	Poliklinik für Zahnerhaltung, Carolinum Dental University Institute gGmbH, Johann-Wolfgang-Goethe University Frankfurt
**Setting**	The courses Restorative Dentistry 1 and 2 take place in the 2nd and 4th clinical semester of the dental medicine programme. Each course runs for 16 semester hours. The students carry out dental treatment under supervision.
**Aim**	Reflection of the treatment session regarding practical skills, theoretical knowledge and doctor-patient communication.
**Feedback giver**	Course tutor (dentist)
**Feedback receiver**	Students in the 2nd and 4th clinical semesters in the dentistry course, treatment courses in Restorative Dentistry 1 and 2
**Feedback material**	Dental treatment carried out by students under supervision with their practical, theoretical and communicative aspects.
**Feedback type**	Oral feedback after patient treatment, formative
**Procedure**	Following dental treatment, once the patient has been discharged, the administrative tasks and documentation are first carried out and signed off together with the students in the appropriate treatment box (patient files, attendance certificate booklet). Subsequently, a 2-5 minute feedback discussion is held regarding the treatment that has just been carried out. Practical skills, theoretical knowledge and doctor-patient communication are discussed. Part of each discussion is the reflection on the treatment session by the course tutor (dentist) as well as by the students. The student then receives suggestions and tips or works out suggestions for improving their practical and communicative skills. In the case of theoretical deficits, students receive tips on where and how they can work on and fix them (learning platform, scripts, literature). Then a written report in the form of keywords is taken down.
**Rationale**	Based on the experience of the author at another location, the feedback discussions for the winter semester 2015/16 were introduced in all clinical courses on restorative dentistry. A previously established grading system was replaced. A decisive factor for this was the students' desire for detailed feedback and an improvement in the learning environment. All course tutors received four hours of training beforehand.
**Literature**	Kurtz, Silverman & Draper: Teaching and Learning Communication Skills in Medicine, 2005<br>Parkes et al., 2013

Attachment 6 to: Thrien C, Fabry G, Härtl A, Kiessling C, Graupe T, Preusche I, Pruskil S, Schnabel K, Sennekamp M, Rüttermann S, Wünsch A. *Feedback in medical education – a workshop report with practical examples and recommendations*. GMS J Med Educ. 2020;37(5):Doc46.
DOI: 10.3205/zma001339

**Experiences**	Based on the experience at the other location and based on the feedback from the students about the intended introduction, there were reservations about the introduction, as this reduced patient time by 30 minutes per day.
**Further development**	The actual acceptance by the students was subsequently recorded as part of the regular evaluation.<br>The approach was established for the long term; there are regular teacher trainings.<br>The literature gives indications that the implementation of a feedback sandwich in written form leads to higher acceptance but not to better performance. This would have to be checked for oral feedback in the form described here. Studies to investigate this issue are planned.

Kommentiert [KC1]: Den Satz verstehe ich nicht, leider fehlt mir das deutsche Original

Attachment 6 to: Thrien C, Fabry G, Härtl A, Kiessling C, Graupe T, Preusche I, Pruskil S, Schnabel K, Sennekamp M, Rüttermann S, Wünsch A. *Feedback in medical education – a workshop report with practical examples and recommendations*. GMS J Med Educ. 2020;37(5):Doc46.
DOI: 10.3205/zma001339
