# Supplementary material for: Feedback in medical education – a workshop report with practical examples and recommendations
Source: GMS J Med Educ. 2020 Sep 15;37(5):Doc46. doi: 10.3205/zma001339 (PMC7499466; doi:10.3205/zma001339)
Supplement: Practical Example General Practice [file JME-37-46-s-007.pdf]

Attachment 7: Practical Example General Practice

Title/keyword of the training	Feedback on patient reports from General Practice attachments
**Author**	Paul Jansen
**Institution**	Formerly Institute of General Practice at the Witten/Herdecke University<br>Currently General Practice Area UKM Münster
**Setting**	In the model curriculum, 6 block courses of General Practice are to be completed over the course of studies in different GP settings. They are divided into sections of two weeks in the beginning. Later two blocks were shortened to one week each. Students receive tasks in a portfolio that consist of preparing patient reports in higher semesters.
**Aim**	Students learn to structure patient contacts and to justify and document diagnostics and treatment.
**Feedback giver**	General Practicioners with teaching and assessment experience
**Feedback receiver**	Students
**Feedback material**	Patient reports from students after internships in GP.
**Manner of feedback**	Written structured evaluation based on a defined expectation horizon with a score. Rating checklist for ticking and with free text comments. Formative feedback with minimum requirements in the 3rd block course, summative, graded feedback in the 4th General Practice block course.
**Procedure**	In the portfolio, participants receive a vignette for structuring patient reports that is based on the SOAP schema (**S**ubjective, **O**bjective, **A**ssessment, **P**lan) for structured patient presentation. The vignette already contains the scaling used for grading in the final block course, as well as a brief description of the learning objective for each task step.<br>Students should derive meaningful differential diagnoses from the patient history, describe a tentative diagnosis and develop a treatment plan based on the actual examination results from the encounter. Possible red flags (information that is indicative of a dangerous progression of an illness) should be taken into account.<br>The subsequent treatment plan should be developed on the basis of recommended guidelines and be justified briefly. Finally, the students should create a short prognosis on the expected progression of the patient.
**Rationale**	Students are in very different GP settings during their block courses. They often see lots of patients but do not process the encounters in a structured manner and only carry out further investigations in single cases. The tasks in the portfolio aim at highlighting and achieving the

Attachment 7 to: Thrien C, Fabry G, Härtl A, Kiessling C, Graupe T, Preusche I, Pruskil S, Schnabel K, Sennekamp M, Rüttermann S, Wünsch A. *Feedback in medical education – a workshop report with practical examples and recommendations*. GMS J Med Educ. 2020;37(5):Doc46.
DOI: 10.3205/zma001339

	learning objectives and ensuring a homogeneous level of performance.<br>Preparing the patient reports supports the gradual, rational development of diagnostic and treatment concepts with the help of the literature. This approach gradually introduces the students to the necessary routines in everyday clinical practice.
**Literature**	1. AMEE Medical Education Guide No. 11 (revised) : Portfolio-based learning and assessment in medical education Maggie Challis Queen's Medical Centre, University of Nottingham, UK<br>2. Portfolios and assessment: meeting the challenge Maggie Challis<br>3. Use of portfolios in early undergraduate medical training Erik W. Driessen Jan van Tarwijk, Jan D. Vermunt & Cees P.M. va der Vleuten<br>4. AMEE Medic al Education Guide No. 24: Portfolios as a method of student assessment M. Friedman, Ben David<br>5. AMEE Guide No. 25: The assessment of learning outcomes for the competent and reflective physician J.M. Shamway, & R.M. Harden<br>6. Developing the teaching instinct. Scottish Council for postgraduate Medical and Dental Education. 1. Feedback. E. A. Hesketh, J. M. Laidlaw
**Experiences**	The portfolio-based approach in the problem-oriented model curriculum was co-developed by students. As the complexity of the tasks increases, the portfolio puts high demands on the students but these are based on the learning objectives of the study regulations. Without structuring the feedback sheet and making the scaling transparent (how many credits can be received for which of the tasks), dissatisfaction with the global rating of the evaluators was high due to the perceived lack of transparency. The feedback form and the clear scaling made acceptance easier.
**Further development**	Constant further development seems necessary, as the students find it increasingly difficult to deal with open and complex questions.

Attachment 7 to: Thrien C, Fabry G, Härtl A, Kiessling C, Graupe T, Preusche I, Pruskil S, Schnabel K, Sennekamp M, Rüttermann S, Wünsch A. *Feedback in medical education – a workshop report with practical examples and recommendations*. GMS J Med Educ. 2020;37(5):Doc46.
DOI: 10.3205/zma001339
