# Supplementary material for: Feedback in medical education – a workshop report with practical examples and recommendations
Source: GMS J Med Educ. 2020 Sep 15;37(5):Doc46. doi: 10.3205/zma001339 (PMC7499466; doi:10.3205/zma001339)
Supplement: Practical Example Emotions [file JME-37-46-s-008.pdf]

Attachment 8: Practical Example Emotions

**Title/keyword of the training**	**Empathy and dealing with emotions**
**Authors**	Tanja Graupe, Claudia Kiessling
**Institution**	University Hospital Munich, Institute for Medical Didactics and Education
**Setting**	Development of a video-based test on empathy and dealing with emotions for formative use in the Medical Curriculum Munich (MeCuM), accompanying the seminars in Medical Psychology in the 3rd pre-clinical semester.
**Aim**	Students learn skills to recognize open and hidden cues and concerns from patients in standardized situations and to select appropriate empathetic strategies in response.
**Feedback giver**	Automated computer-based feedback based on a theoretical framework and on an expert panel
**Feedback receiver**	Medical students
**Feedback material**	Short trigger videos of student/doctor-patient encounters which contain an emotional cue or concern of the patient followed by predefined behavioral alternatives to be evaluated for adequateness by the students
**Feedback type**	Computerized feedback, formative, based on experts and theory
**Procedure**	In a first step, doctors were asked in interviews to describe everyday clinical situations in which the handling of emotions played a role. In a next step, these descriptions were qualitatively analyzed, converted into scripts in a multi-stage process and videotaped with medical students, doctors and simulated patients. 18 situations were generated, each with two short videosequences, which were transferred to the online-based learning platform CASUS (http://lmu.casus.net/). These 18 situations were then validated in workshop of experts and 12 situations were included in a final test version for students.<br>In CASUS, students are asked after each videosequence to use a slider to assess the appropriateness of five given behavioral alternatives in relation to the situations shown. The behavioral alternatives were developed and validated beforehand based on current theories and an expert panel.<br>After the students have evaluated the alternatives, they receive automated written feedback in CASUS on how the expert panel assessed the appropriateness of the behavioral alternatives and which theoretical communication strategies underlie the behavioral alternatives.

Attachment 8 to: Thrien C, Fabry G, Härtl A, Kiessling C, Graupe T, Preusche I, Pruskil S, Schnabel K, Sennekamp M, Rüttermann S, Wünsch A. *Feedback in medical education – a workshop report with practical examples and recommendations*. GMS J Med Educ. 2020;37(5):Doc46.
DOI: 10.3205/zma001339

**Rationale**	The test format is based on the principles of the Situational Judgment Test*. The predefined behavioral alternatives are based on the "Verona coding definitions of emotional sequences (VR-CoDES)"**.<br>In a standardized situation, students learn to recognize sequences which involve patient's emotions and react appropriately, ideally in an empathetic way. The topic can be taught to a large number of students through the computer-based approach.
**Literature**	*Patterson F., Ashworth V., Zibarras L., Coan P., Kerrin M., O'Neill P. (2012). Evaluations of Situational Judgment Tests to assess non-academic attributes in selection. Medical Education, 46 (9), 850–868.<br>** Zimmermann C, Del Piccolo L, Bensing J, et al. Coding patient emotional cues and concerns in medical consultations: The Verona coding definitions of emotional sequences (VR-CoDES). Pat Educ Couns 2011; 82:141–148.
**Experiences**	The test is still in the testing phase.<br>A first run with medical students showed good functionality and acceptance among medical students. In terms of feedback, the students asked for an assessment of the communication strategies by experts and an explanation of the theoretical reasoning.
**Further development**	Testing the test in regular classes with over 900 students per year.<br>Questions posed:<br>1. Does a video and computer-based test with feedback accompanying the course increase the acceptance of communication lessons among medical students in the pre-clinic phase?<br>2. Does the test improve communication skills during the course?

Attachment 8 to: Thrien C, Fabry G, Härtl A, Kiessling C, Graupe T, Preusche I, Pruskil S, Schnabel K, Sennekamp M, Rüttermann S, Wünsch A. *Feedback in medical education – a workshop report with practical examples and recommendations*. GMS J Med Educ. 2020;37(5):Doc46.
DOI: 10.3205/zma001339
