# Supplementary material for: Feedback in medical education – a workshop report with practical examples and recommendations
Source: GMS J Med Educ. 2020 Sep 15;37(5):Doc46. doi: 10.3205/zma001339 (PMC7499466; doi:10.3205/zma001339)
Supplement: Practical Example Standardized Patients (SPs) [file JME-37-46-s-009.pdf]

Attachment 9: Practical Example Standardized Patients (SPs)

**Title/keyword of the training**	**Feedback training for SPs**
**Author**	Christian Thrien
**Institution**	Cologne Interprofessional Skills Lab and Simulation Center, University of Cologne
**Setting**	Feedback training for SPs used in courses of the clinical phase of Medical Education. The courses include a wide variety of settings, from taking a patient's medical history and physical examination to the delivery of bad news.
**Aim**	SPs should provide adequate and constructive feedback on medical consultation skills and the handling of patients by students
**Feedback provider**	Simulated patients
**Feedback receiver**	Simulated patients
**Feedback material**	Improvised scenes of two SPs each in a non-medical context, after brief descriptions of roles and situations that contain conflicts
**Feedback type**	Formative feedback, experience-oriented
**Procedure**	First, the participants' prior knowledge and experiences regarding feedback and communication are activated and reflected on.<br>Afterwards, short role descriptions are used to improvise scenes in a non-medical context. The participants then give each other feedback and receive feedback on the feedback from observing participants and the trainer.<br>In this task, the SPs do not use objective standards as guidance but rely on their personal feelings. They differentiate between their perception (what did I see/hear?), their interpretation of this perception (what do I suspect was meant/thought etc.?) and their subsequent, including emotional, reaction (how do I feel?)<br>Preparatory exercise: An SP chooses a non-neutral posture and the group describes their observation in three steps using sentence starts provided:<br>"I see ..." (purely descriptive, for example: "... a man with his head bowed, his hands supporting his chin" etc.)<br>"I suspect ..." (interpreting, for example: "... the man is exhausted and sad.")<br>"I feel ..." (describing your own emotional reaction, for example: "... encouraged me to ask him how he is doing")<br>Particular emphasis is placed on the distinction between the first and the other two aspects.<br>The distinction is the prerequisite for an I-statement.
**Rationale**	An I-statement according to Schulz von Thun requires clarification from the feedback provider beforehand in which situation they reacted to which signals from their counterpart and how. Including

Attachment 9 to: Thrien C, Fabry G, Härtl A, Kiessling C, Graupe T, Preusche I, Pruskil S, Schnabel K, Sennekamp M, Rüttermann S, Wünsch A. *Feedback in medical education – a workshop report with practical examples and recommendations*. GMS J Med Educ. 2020;37(5):Doc46.
DOI: 10.3205/zma001339

	this in the feedback makes it possible to address situations in which communication was not ideal and to stimulate reflection on it by the recipient of the feedback without judging or condemning them or their behavior.<br>The feedback procedure should prevent this in two ways.<br>The feedback is always subjective. There is no objective standard for the SP. So there are no mistakes.<br>The I-statement, with a clear separation of perception and interpretation and the identification of this in the feedback, always makes it clear that the interpretation is carried out by the feedback provider (SP). There is no claim to generality.
**Literature**	Schulz von Thun, Friedemann: Miteinander reden 1 – Störungen und Klärungen, Rowohlt Taschenbuch, Reinbek bei Hamburg 1981
**Experiences**	This approach enables feedback that is critical and respectful at the same time. However, critical feedback is often avoided in teaching.<br>A challenge for SP lies in having to simulate, to observe their respective counterpart and their own emotional reactions at the same time in order to reflect them in the feedback. A short preparation time and questionnaires with leading questions based on the learning objectives help with the structuring.
**Further development**	Question: How can SPs be encouraged to implement critical feedback.

Attachment 9 to: Thrien C, Fabry G, Härtl A, Kiessling C, Graupe T, Preusche I, Pruskil S, Schnabel K, Sennekamp M, Rüttermann S, Wünsch A. *Feedback in medical education – a workshop report with practical examples and recommendations*. GMS J Med Educ. 2020;37(5):Doc46.
DOI: 10.3205/zma001339
